# Facteurs prédictifs de descellement aseptique des prothèses totales cimentées de la hanche

**DOI:** 10.11604/pamj.2016.24.260.8164

**Published:** 2016-07-20

**Authors:** Rateb Kochbati, Hedi Rbai, Marouene Jlailia, Hassen Makhlouf, Abderrazak Bouguira, Med Samir Daghfous

**Affiliations:** 1 Service de Chirurgie Orthopédique et Traumatologique, Institut Kassab d’orthopédie, Tunis, Tunisie

**Keywords:** Hip, total arthroplasty, aseptic loosening, risk factor, Hip, total arthroplasty, aseptic loosening, risk factor

## Abstract

**Introduction:**

Le descellement aseptique constitue la principale complication à long terme et signe la faillite de la prothèse totale de hanche. Les causes de descellement aseptique sont multiples et souvent intriquées. Le mal positionnement des implants reste le facteur le plus incriminé. D’autres facteurs liés au patient et à la prothèse prédisposent également au descellement mais à des degrés divers.

**Méthodes:**

A travers une étude rétrospective portant sur 64 descellements aseptiques de prothèse totale de hanche, nous avons tenté d’individualiser les facteurs de descellement lié au patient, au type d’implant et à la technique chirurgicale et d’en dégager les recommandations visant à minimiser ce risque. Il s’agissait d’une étude rétrospective analytique portant sur 64 descellements aseptiques. La classification utilisée est celle de la Société Française de Chirurgie Orthopédique et Traumatologique.

**Résultats:**

La moyenne d’âge au moment de la première arthroplastie était de 40 ans. Elle était de 62 au moment du descellement. La tige type Charnley a été implantée dans 55 cas, celle de type Muller dans 9 cas. La pièce cotyloïdienne a été bien positionnée dans 69% des cas avec une inclinaison moyenne de 47,8°. Les tiges étaient remplissantes dans 86% des cas avec un cimentage Grade A dans 60% des cas. Le délai moyen de la survenue du descellement était de 12 ans. 72% des prothèses avaient une survie supérieure à 10 ans. L’analyse statistique des résultats a individualisé les facteurs de risque du descellement que sont: L’âge, l’indexe de masse corporelle, le niveau d’activité, l’inclinaison de la cupule, le déport fémoral et la qualité du cimentage.

**Conclusion:**

Une réduction significative des descellements aseptiques des prothèses totales de hanche ne pourra être obtenue que par une plus grande rigueur dans la sélection des patients, une plus grande sûreté dans l’acte technique et un meilleur choix de l’implant à poser.

## Abstract (en)

**Introduction:**

Aseptic loosening is the main long-term complication and indicates total hip replacement failure. There are many and often intricate causes for aseptic loosening. Incorrect placement of the implants is the most incriminated factor. Other patient and implant related factors may also predispose to loosening but to various degrees.

**Methods:**

We conducted a retrospective study of 64 cases with aseptic loosening of total hip prosthesis to individuate patient, implant type and surgical technique related factors leading to aseptic loosening and to provide recommendations to minimize this risk.

**Results:**

This was an analytic retrospective study of 64 cases with aseptic loosening. The classification is that used by the French Society of Orthopedic Surgery and Traumatology. The average age of the patients at the time of the first arthroplasty was 40 years, 62 years at the time of loosening. Charnley stem was implanted in 55 cases, Muller stem in 9 cases. Acetabular component was well positioned in 69% of cases with an average inclination of 47.8 °. The stems were canal-filling in 86% of cases with Grade A cementation in 60% of cases. The mean time for loosening onset was 12 years. 72% of the prostheses had a survival longer than 10 years. Statistical analysis of the results identified risk factors leading to loosening, such as: age, body mass index, level of activity, cup inclination, femoral offset and cementation quality.

**Conclusion:**

A significant reduction in aseptic loosening of total hip prostheses may only be achieved by a more rigorous selection of patients, a greater security in the technical act and a better choice of the implant to be placed

## Introduction

L’arthroplastie totale de la hanche a connu un essor remarquable en Tunisie depuis son introduction à la fin des années 80. Ses indications se sont élargies et touchent des patients de plus en plus jeunes et actifs. Malheureusement, les implants posés ainsi que les couples de frottement ne sont pas éternels. Leur longévité ne dépasse pas les vingtaines d’années dans les meilleurs des cas [1]. Le descellement aseptique, uni ou bipolaire, constitue la principale complication à long terme et signe la faillite, du moins radiologique, de la prothèse totale de hanche. Les causes de descellement aseptique sont multiples et souvent intriqués. Le mal positionnement des implants reste le facteur le plus incriminé [2]. D’autres facteurs liés au patient et à la prothèse prédisposent également au descellement mais à des degrés divers. A travers une étude rétrospective portant sur 64 descellements aseptiques, uni et bipolaire, de prothèse totale de hanche, nous tenterons d’individualiser les facteurs de descellement liés au patient, au type d’implant et à la technique chirurgicale et d’en dégager les recommandations visant à minimiser ce risque.

## Méthodes

Il s’agit d’une étude analytique rétrospective portant sur 56 patients et 64 hanches, suivis à l’Institut National d’Orthopédie MT Kassab, entre Janvier 2000 et Décembre 2013 pour un descellement aseptique d’une prothèse totale cimentée de la hanche (PTH).

**Critères d’inclusion:** Les patients présentant un descellement aseptique uni ou bipolaire symptomatique ou asymptomatique d’une PTH cimentée, et les patients ayant eu un couple de frottement inox polyéthylène.

**Critères d’exclusion:** Les patients ayant un syndrome inflammatoire biologique, et les patients ayant un prélèvement bactériologique peropératoire positif.

**Données cliniques:** Nous avons noté pour chaque dossier le sexe, l’âge des patients au moment de la première arthroplastie de la hanche et au moment de l’apparition du descellement, les antécédents médicaux et chirurgicaux, le niveau d’activité, l’étiologie initiale amenant à l’arthroplastie. Nous avons également relevé la voie d’abord utilisée, le type de la prothèse totale de la hanche. L’examen recherchait une attitude vicieuse, évaluait la trophicité musculaire, la stabilité en appui monopodal et recherchait une boiterie. Les amplitudes articulaires ont été mesurées. L’examen était toujours comparatif. Le poids et la taille ont été consignés et l’indice de masse corporelle (IMC) calculé. Le niveau d’activité a été évalué par le score de Charnley [3]. Au terme des données anamnestiques et cliniques, le score de Postel Merle D’Aubigné [4] a été établi. Il renseignait sur le degré de retentissement fonctionnel de la coxopathie.

### Données de l’imagerie

**Figure 1:**
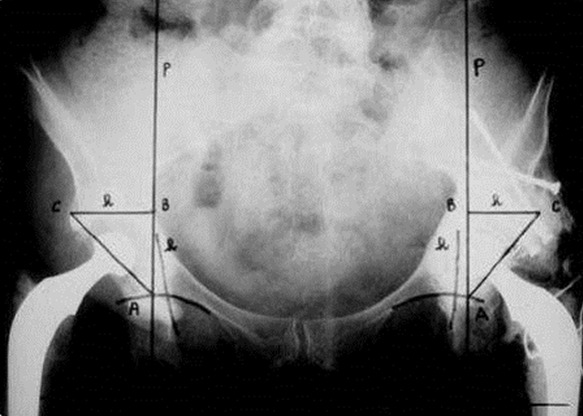
Positionnement du cotyle selon Ranawat

**Figure 2:**
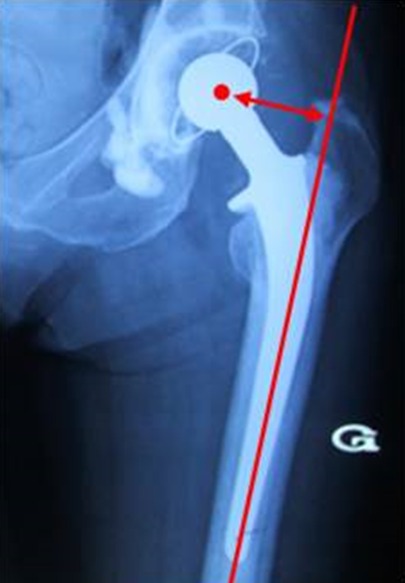
Calcul du déport femoral

**Radiographie post opératoire:** Les radiographies postopératoires immédiates du bassin de face et de la hanche opérée de profil permettaient d’évaluer plusieurs paramètres:

Le positionnement de la pièce cotyloïdienne (Figure 1) a été évalué selon les critères de Ranawat [5]. L’inclinaison de la pièce cotyloïdienne. Le déport ou offset fémoral (Figure 2) [6]. Calibrage de la tige fémorale [7]. Qualité de cimentage de la tige fémorale [8].

**Figure 3:**
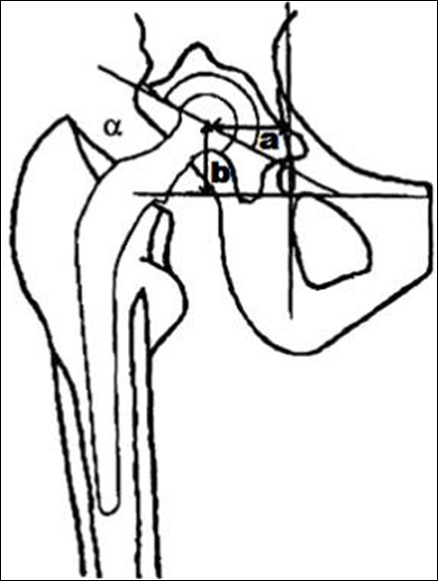
Appréciation de la migration de la cupule: une diminution de (a) indique une protrusion, une augmentation de (b) indique une ascension, une variation de (α) indique une rotation

**Figure 4:**
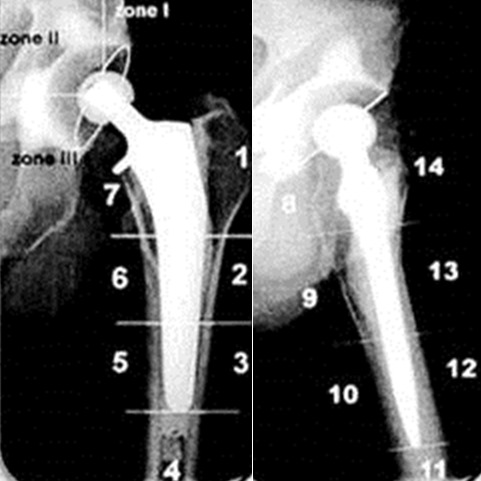
Zones cotyloïdiennes de De Lee et fémorales de Gruen

**Figure 5:**
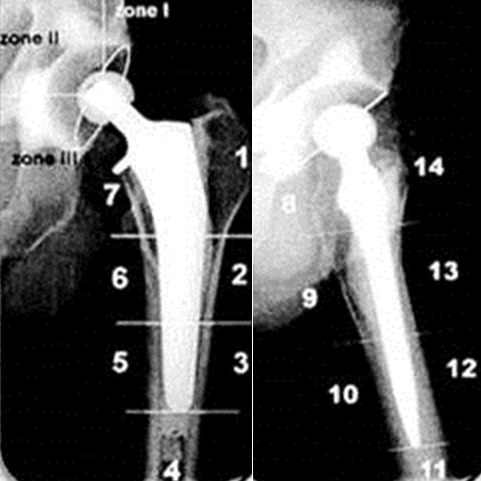
Usure du polyéthylène: en l’absence d’usure, le milieu du segment joignant les deux extrémités de la cupule et le centre de la tête sont superposables; lorsqu’ils ne le sont plus, la distance entre ces deux points quantifie l’usure

**Radiographie au dernier recul:** Les radiographies de bassin de face et de hanche de profil permettaient de poser le diagnostic de descellement et de le classer selon Harris [9]. Elles permettaient également de rechercher et de classer l’ostéolyse ainsi que les ossifications hétérotopiques. Diagnostic radiologique du descellement: Deux éléments signent le descellement: La migration des implants (Figure 3) et la présence d’un liseré évolutif total ou partiel classiquement authentifié lorsqu’il atteint 2 mm. Au niveau du cotyle, le liseré est noté en fonction des trois zones classiques de De Lee [10]. Au niveau du fémur, le descellement est classé selon les zones de Gruen [11] (Figure 4). Classification des descellements cotyloïdiens et fémoraux: La classification utilisée est celle de la Société Française de Chirurgie Orthopédique et Traumatologique (SOFCOT) [12]. Classification des ossifications hétérotopiques selon Brooker [13]. Usure du polyéthylène (Figure 5).

**Tomodensitométrie:** La tomodensitométrie (TDM) n’était pas systématique. Elle a été réalisée dans les cas ou le diagnostic n’était pas certain.

**Scintigraphie osseuse:** La scintigraphie au technétium 99 a été pratiquée dans les cas douteux de descellement. La scintigraphie aux leucocytes marqués a été réalisée dans les suspicions de descellements septiques avec une clinique et une biologie non concluantes.

## Résultats

**Données épidémiologiques:** La série était composée de 31 hommes et 25 femmes avec un sex-ratio de 1,24. La moyenne d’âge au moment de la première arthroplastie était de 40 ans avec une médiane de 42 ans et des extrêmes de 17 et 62 ans. 59% des patients étaient âgés de plus de 40 ans. La moyenne d’âge au moment du descellement était de 62 ans avec des extrêmes de 24 et 78 ans. Le délai moyen de survenu du descellement était de 12 ans avec des extrêmes de 3 et 25 ans. 72% des prothèses avaient une survie supérieure à 10 ans. 8 patients ont eu une arthroplastie descellée des deux hanches, le côté droit a été objectivé dans 32 cas (57%).

**Chirurgie première:** Une coxarthrose idiopathique a été retenue chez 19 patients soit 30% des cas. La fracture du col du fémur vient en deuxième place, ex aequo avec les coxites inflammatoires, toutes les deux observées dans 13 cas (Tableau 1). La voie antérolatérale de Hardinge a été réalisée dans 52 cas (81%), celle de Moore dans 12 cas (19%). Le couple utilisé était le polyéthylène-inox. La taille moyenne de la pièce cotyloïdienne était de 50 mm avec des extrêmes de 44 et 54. Le diamètre de la tête implantée était de 22 mm (Prothèse monobloc) dans 53 cas, et de 28 mm (Prothèse modulaire) dans 11 cas. La taille moyenne de la tige implantée était de 4 avec des extrêmes de 1 et 7. La tige type Charnley (Tige non anatomique) a été implantée dans 55 cas, celle de type Muller (Tige anatomique à appui métaphysaire) dans 9 cas.

**Tableau 1:** Répartition selon l’étiologie

Etiologie	Cas	Poucentage (%)
Coxarthrose primitive	19	30
Fracture col du fémur	13	20
Coxite inflammatoire	13	20
Ostéonécrose	12	19
Dysplasie de hanche	4	6
Coxarthrose post-traumatique	3	5

**Données anamnestiques et cliniques:** La boiterie était présente dans tous les cas. Le poids moyen était de 80,64 Kg avec des extrêmes de 70 et 90 Kg. L’IMC moyen était de 26,4 Kg/m^2^ avec des limites allant de 21 à 32,7 Kg/m^2^. 39,1% de patients avaient un poids normal, 42,2% étaient en surcharge pondérale et 18,7% avaient une obésité. 7,8% des patients présentaient des facteurs ne permettant pas une locomotion normale. Le score moyen de la douleur était de 2,5/6, celui de la mobilité était de 4,6/6 et celui de la stabilité était de 3,5/6. Ainsi, le score PMA moyen était de 10,6/18 avec des extrêmes de 8 et 15 (Tableau 2).

**Tableau 2:** Répartition selon le score PMA

Score	PMA
Très bon (18)	0
Très bon (17)	0
Bon (15-16)	2
Passable (13-14)	6
Médiocre (9-12)	44
Mauvais (<9)	12

### Imagerie

**Radiographie post opératoire:** La pièce cotyloïdienne a été implantée correctement selon les critères de Ranawat dans 44 cas soit 69% des cas. L’angle moyen d’inclinaison était de 47,8° avec des extrêmes de 33 et 60°. 28 cupules étaient dans la fourchette de 40 à 50%. Le déport fémoral a été rétablit dans 50 cas (78%). 11 tiges fémorales étaient varisées soit 11% des cas. 5 tiges étaient enfoncées et 2 suspendues. Les tiges étaient remplissantes dans 55 cas (86%). Selon Barrack, 38 tiges étaient de grade A, 14 de grade B et 12 de grade C.

**Radiographie au dernier recul:** Concernant la pièce cotyloïdienne, le liséré de descellement était localisé dans les 3 zones de De Lee dans 52 cas sur 58 descellements cotyloïdiens soit 89%. La cupule était en place dans 16 cas (25%), le déplacement était supérieur dans 17 cas (26%), interne dans 11 cas (17%) et supéro-interne dans 20 cas (32%). Le déplacement rotatoire a été observé dans 8 cas avec une luxation ou une subluxation de la cupule. Aucun descellement au stade I de la SOFCOT n’a été noté. Quant à la pièce fémorale, Le liseré de descellement a été visualisé dans tous les zones de Gruen uniquement dans 3 cas. La tige fémorale était en place dans 37 cas sur 64 descellements (58%). Un enfoncement a été noté dans 5 cas, une varisation dans 18 cas et les deux associés dans 3 cas. Enfin, un seul cas de cassure de la tige a été noté. Le stade I de la SOFCOT a été noté dans 34 cas (53%). Une usure du polyéthylène a été notée dans 38 cas. Toutes les cupules usées étaient descellées. Concernant les ossifications hétérotopiques, le stade I de Brooker a été noté dans 26 cas, le stade II dans 11 cas. Les autres hanches ne présentaient pas d’ossifications hétérotopiques.

**Tomodensitométrie:** La TDM a été pratiquée chez 7 patient afin d’évaluer le capital osseux acétabulaire et fémorale. Elle a été couplée à une angiographie dans 2 cas de protrusion cupulaire importante. Dans les deux cas, il n’y avait pas de contact avec les vaisseaux iliaques.

**Scintigraphie osseuse:** La scintigraphie aux leucocytes marqués a été pratiquée chez un seul patient qui présentait un descellement précoce. Elle montrait une absence de fixation significative éliminant ainsi une origine septique.

### Analyse uni variée des résultats

**Facteurs liés au patient:** Le sexe ne constitue pas un facteur prédictif de descellement (p = 0,8). On a noté que le taux de descellement diminue significativement à partir de 50 ans. La valeur de p étant égale à 0.04, le jeune âge constitue donc un facteur prédictif de descellement. le jeune âge constitue donc un facteur prédictif de descellement. Selon nos données, le poids ainsi que l’IMC ne constituent pas un facteur de risque de descellement (p = 0,15). On a noté que les courbes de survie des PTH sur coxite inflammatoire et l’ostéonécrose de la tête fémorale s’éloignent nettement des celles de la coxarthrose et de la fracture cervicale. Ce risque, plus élevé sans être significatif (p = 0,11), peut être expliqué par la mauvaise qualité osseuse dans ces deux premières pathologies. Dans notre série, le niveau d’activité est un facteur prédictif de descellement (p = 0,01). La survie est meilleure au stade A de Charnley.

**Facteurs liés au type d’implant:** Aucune différence significative n’a été notée entre la tige Charnley et la tige Muller (p = 0,77). Selon l’étude statistique, la survie des prothèses est meilleure pour les têtes de 28 mm sans pour autant y avoir une différence significative (p = 0,12).

**Facteurs liés à la technique chirurgicale:** Aucune différence significatives n’a été notée entre les voies antérolatérale et postérieure. Le non rétablissement du centre de la hanche ne semble pas affecter la survie (p = 0,3). En revanche, la durée de vie d’une prothèse dans notre série est meilleure si l’angle d’inclinaison du cotyle se situe entre 40° et 50° (p = 0,01). L’angle d’inclinaison de la pièce cotyloïdienne constitue ainsi un facteur déterminant de descellement. Le taux de descellement est corrélé au positionnement de la tige dans le plan frontal. Une diminution du déport fémoral augmente significativement le risque de descellement prothétique (p = 0,001). la survie semble varier en fonction du degré de remplissage du fémur, mais le test statistique est non significatif (p = 0,8). La qualité de cimentage constitue un important facteur de descellement avec un p = 0,001.

### Analyse multi variée des résultats

**Facteurs épidémiologiques:** L’âge jeune des patients et le niveau d’activité constituent les facteurs épidémiologiques influençant la survie de la prothèse en étude univariée. Lorsqu’on étudie la survie en fonction des facteurs épidémiologiques regroupés, on remarque que l’indice de masse corporelle retentit sur la survie de la prothèse conjointement avec l’âge et le niveau d’activité.

**Facteurs liés à la prothèse:** Notre série, les facteurs liés à la prothèse n’agissent pas sur la survie de cette dernière en étude univariée. Il en est de même en étude multivariée.

**Facteurs liés à la chirurgie:** L’inclinaison de la pièce cotyloïdienne, le déport fémoral et la qualité de cimentage de la tige constituent les facteurs déterminant de descellement.

## Discussion

### Facteurs prédictifs du descellement aseptique

#### Facteurs liés au patient

**Le sexe:** Plusieurs études ont étudié l’influence du sexe sur la survie des prothèses totales de hanche [14–17]. Le sexe masculin est incriminé dans le descellement précoce de deux façons: Les hommes sont généralement plus lourds et plus actifs que les femmes du même âge [18]. Ils bénéficient également d’une intervention à un âge plus jeune que les femmes; 67 ans versus 70 ans [16]. Dans notre série, le sexe ne constituait pas un facteur prédictif de descellement.

**L’âge:** Dans notre série, l’âge constitue un facteur prédictif de descellement aseptique. Ce résultat concorde avec celui des différentes publications sur le sujet [16, 19].

**L’IMC:** Plusieurs auteurs a démontré que le poids est un facteur non négligeable dans la genèse d’un descellement aseptique [16, 18, 20]. Cependant, d’autres études n’ont objectivé aucune augmentation du risque de descellement chez les patients obèses malgré l’augmentation de la charge articulaire chez ces patients [21, 22]. Dans notre série, l’IMC n’influence pas à lui seul la survie de la prothèse mais il agit en association avec d’autres facteurs pour favoriser le descellement.

**L’étiologie:** L’influence de l’étiologie initiale sur la survie des prothèses totales de la hanche est variable dans la littérature. Dans notre série, nous n’avons pas trouvé de différence significative entre les patients ayant une polyarthrite rhumatoïde et ceux qui ont une coxarthrose primitive.

**Le niveau d’activité:** Dans notre série, le niveau d’activité, apprécié par le score de Charnley, représente un facteur prédictif de descellement aseptique (p = 0,01). Ceci concorde avec les résultats de plusieurs études [15, 16, 18, 23, 24]. Cependant, il n’y a pas de consensus définissant le niveau d’activité qui doit être entrepris par les patients opérés pour PTH [25]. Les activités en décharge, telles que la natation et le cyclisme sont toujours recommandées.

#### Facteurs liés au type d’implant

**Le type d’implant:** Devant la diversité des implants utilisés, il est difficile d’étudier les différents designs des prothèses et les traitements de surfaces de manière indépendante. Ainsi, des conclusions de la comparaison des mérites respectifs des diverses prothèses mises sur le marché sont difficiles à tirer.

**Le diamètre de la tête:** Les avis des auteurs divergent quant au rôle du diamètre de la tête dans la survenue de descellement aseptique [26].

Le ciment: les fractures du ciment acrylique produisent des particules qui selon certains auteurs sont la principale cause de descellement à long terme des implants fémoraux cimentés [27]. Le polyéthylène: le polyéthylène de dernière génération a une usure plus lente. Les débris occasionnés sont de plus petite taille facilitant leur phagocytose. Néanmoins, ce matériau ne peut empêcher la survenue d’ostéolyse et donc à terme le descellement [28].

#### Facteurs liés au type d’implant

**La voie d’abord:** Les voies d’abord les plus utilisées dans l’arthroplastie totale de hanche sont les voies antérieure, antérolatérale (Hardinge) et postérieure (Moore). Aucune étude n’a comparé l’impacte de ces approches sur le descellement prothétique. Concernant la voie mini invasive, les avis sont partagés et les détracteurs de cette technique sont nombreux.

**Les paramètres de la cupule:** La restitution du centre de la hanche prothésée est primordial voire vitale pour la longévité de la prothèse [29]. Concernant l’inclinaison cupulaire, la majorité des auteurs préconisent une valeur comprise entre 40 et 50° [30]. Autre élément non moins important est l’antéversion de la cupule qui doit être entre 0 et 20° [31]. Un dernier paramètre à prendre en compte est le conflit fémoro-acétabulaire. En effet, le contact répétitif du col sur le rebord cotyloïdien entraîne une augmentation des contraintes transmises à l’interface os-ciment. Ces contraintes réitérées sont à terme nocifs pour la fixation de la cupule mais leur rôle dans le descellement reste très discuté.

**Les paramètres de la tige:** La position de la tige fémorale conditionne sa longévité [32]. Dans le plan frontal, le varus est une position à éviter [33]. Dans notre série, le varus fémoral constituait un facteur prédictif de descellement. Cette position dans le plan frontal influe également sur la restitution du déport fémoral. Ce dernier constitue un élément clé dans la survie d’une prothèse totale de la hanche [34]. Au final, la tige doit se situer dans l’axe du fémur en évitant tout conflit avec les corticales. Une antéversion de 15° est recherchée et le cintre cervico-obturateur doit être parfaitement restitué [32]. Autre élément à considérer: le calibrage de la tige. Un sous calibrage de la tige favoriserait l’enfoncement précoce, le stress shielding et la modification du déport fémoral [35].

**La qualité du cimentage:** Une bonne technique de cimentage constitue un garant d’une meilleure longévité de la prothèse [36].

**Le centre et le chirurgien:** Le type de l’hôpital et l’expérience du chirurgien peuvent influencer la survie des arthroplasties. Fender [37] a montré qu’une faible expérience est associée à un risque accru de révision.

## Conclusion

Les reprises des prothèses descellées sont souvent des actes longs, hémorragiques, pouvant nécessiter des reconstructions difficiles et mal supportées chez le sujet âgé. De ce fait, l’individualisation des facteurs incriminés dans le descellement aseptique permettrait une amélioration de la technique opératoire et une diminution à long terme du taux du descellement. Le rôle du chirurgien apparaît essentiel car il doit s’efforcer de redonner à la hanche une architecture la plus proche de la normale afin de permettre un meilleur jeu articulaire et rétablir une balance musculaire optimale. Ceci suppose que le centre de rotation soit situé en son point électif et que le bras de levier externe soit rétabli. En effet, d’après notre étude, les deux paramètres majeurs de descellement sont le positionnement cupulaire et la qualité de cimentage. Les autres paramètres liés à l’acte chirurgical sont tout aussi importants à planifier et à prendre en compte. Un mauvais calibrage ou une mauvaise orientation de la tige sont préjudiciables à moyen et long termes. Tout ceci est directement lié à l’expérience du chirurgien et à la qualité de l’encadrement dans la structure hospitalière. Quels que soient ces alternatives, une réduction significative des descellements aseptiques ne pourra être obtenue que par une plus grande rigueur dans la sélection des patients, une plus grande sûreté dans l’acte technique et par un meilleur choix de l’implant à poser. Les objectifs de cette chirurgie doivent toujours rester raisonnables.

### Etat des connaissances actuelles sur le sujet

- Les reprises des prothèses descellées sont souvent des actes longs, hémorragiques, pouvant nécessiter des reconstructions difficiles;
- le rôle du chirurgien apparaît essentiel car il doit s’efforcer de redonner à la hanche une architecture la plus proche de la normale afin de permettre un meilleur jeu articulaire et rétablir une balance musculaire optimale;
- Les causes de descellement aseptique sont multiples et souvent intriqués.

### Contribution de notre étude à la connaissance

- D’après notre étude, les deux paramètres majeurs de descellement sont le positionnement cupulaire et la qualité de cimentage;
- La cupule doit être insérée en position anatomique en se référant au ligament transverse;
- Améliorer les techniques de cimentage pour obtenir la meilleure interpénétration du ciment dans l’os est la seule garantie d’une bonne fixation primaire.
